# Supplementary figures and images for: Cyclic di-GMP as an antitoxin regulates bacterial genome stability and antibiotic persistence in biofilms
Source: eLife. 2024 Oct 4;13:RP99194. doi: 10.7554/eLife.99194 (PMC11452175; doi:10.7554/eLife.99194)

Figure 5B

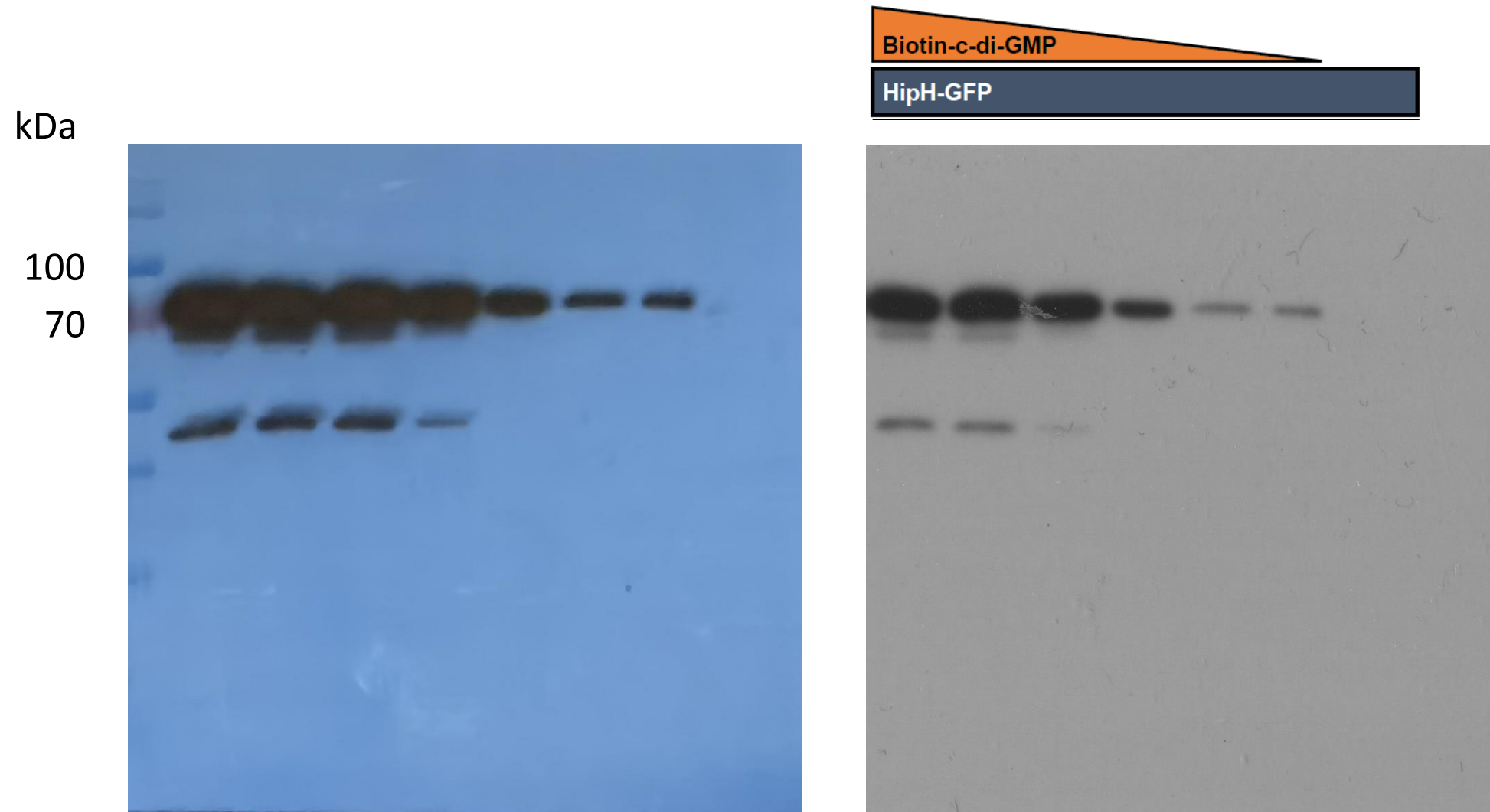

Figure 5C

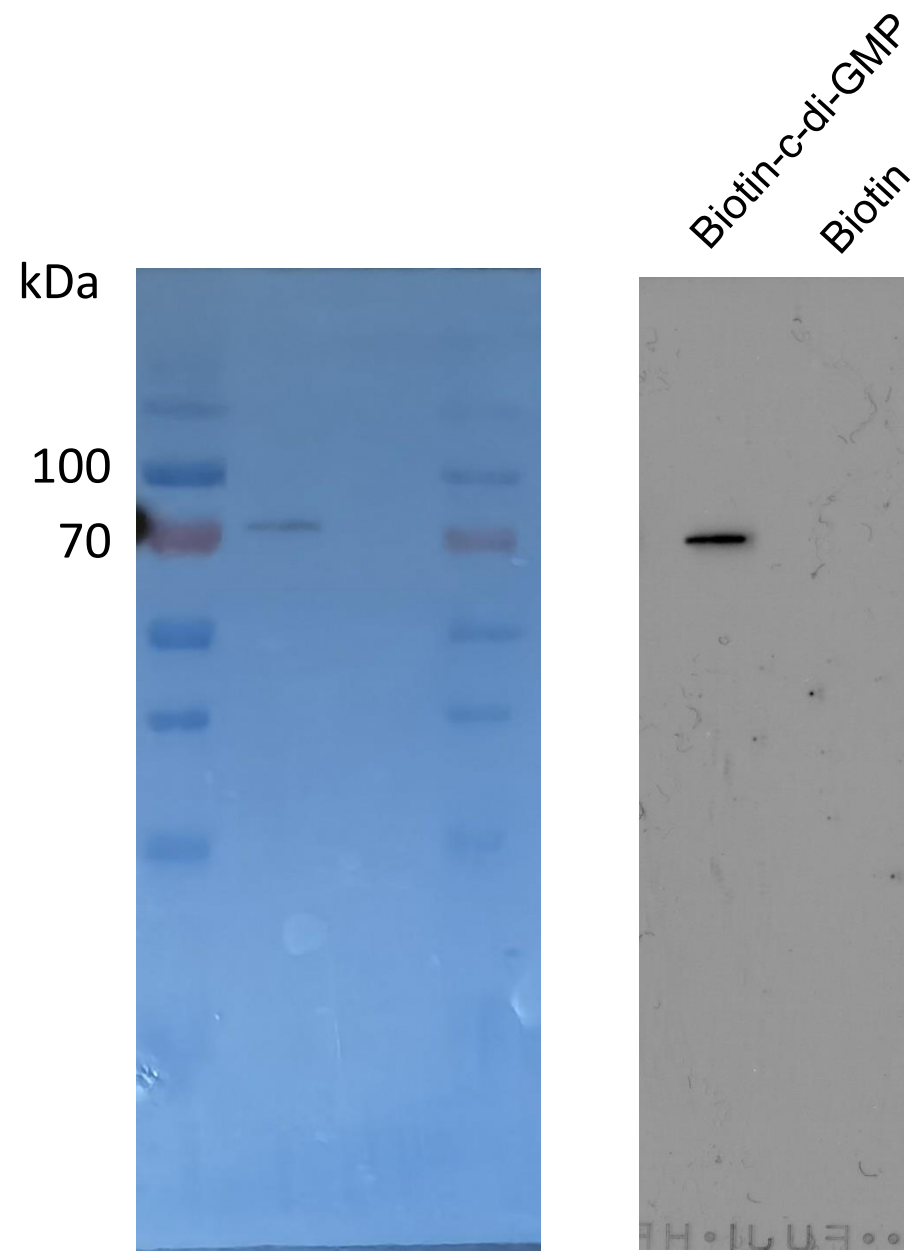

Figure 5D

kDa

100  
70  
55  
40  
35  
25

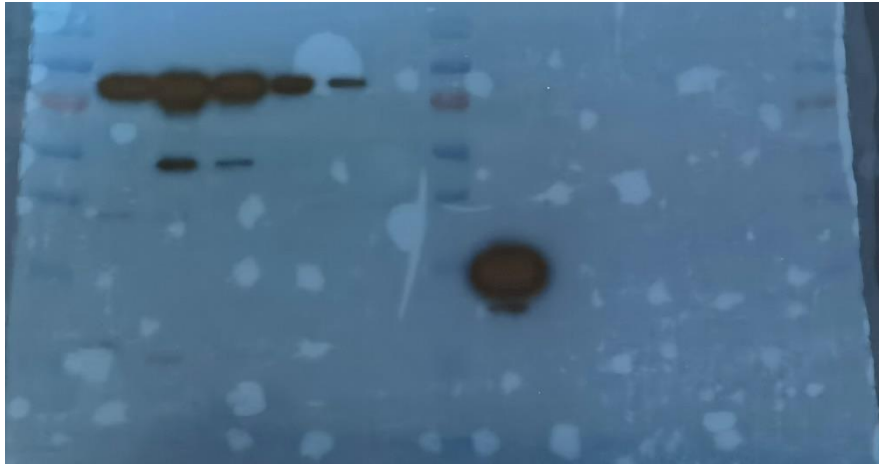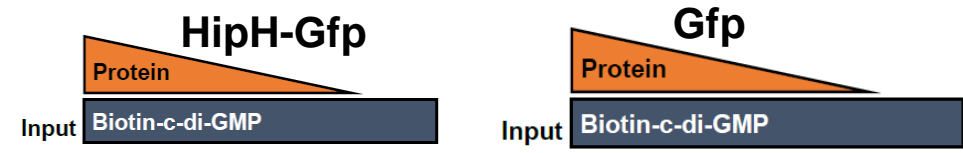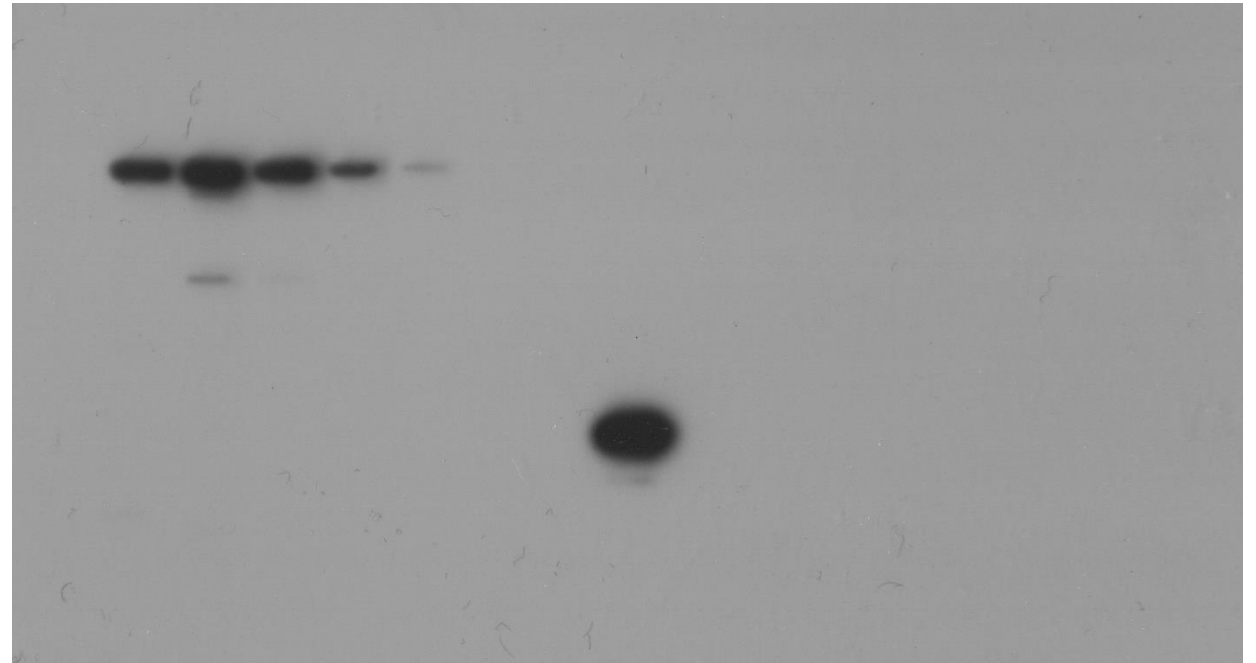

Figure 5E

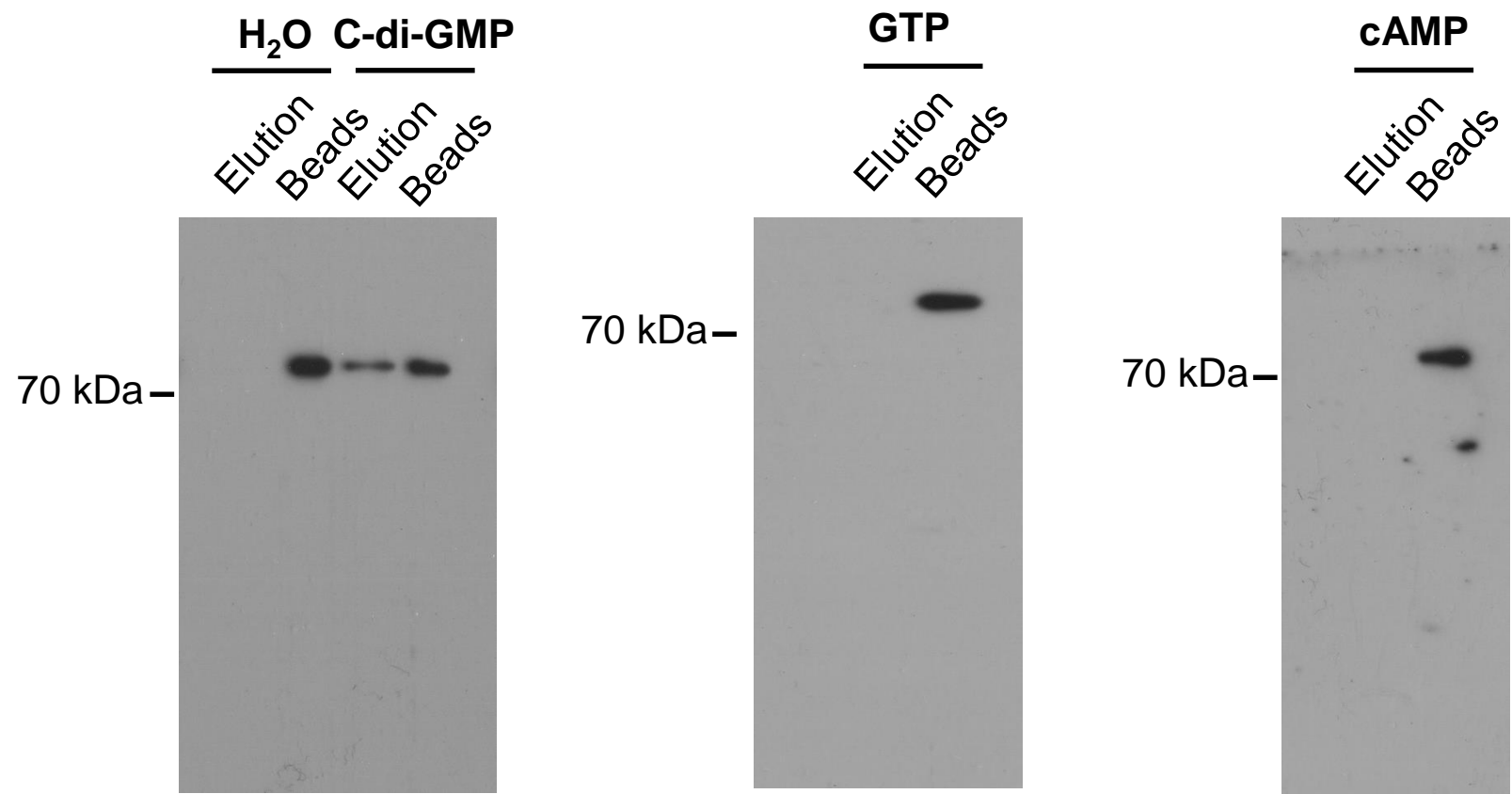

Supplement: Figure 5—source data 1. [file elife-99194-fig5-data1.pdf]

Figure 5—figure supplement 1A

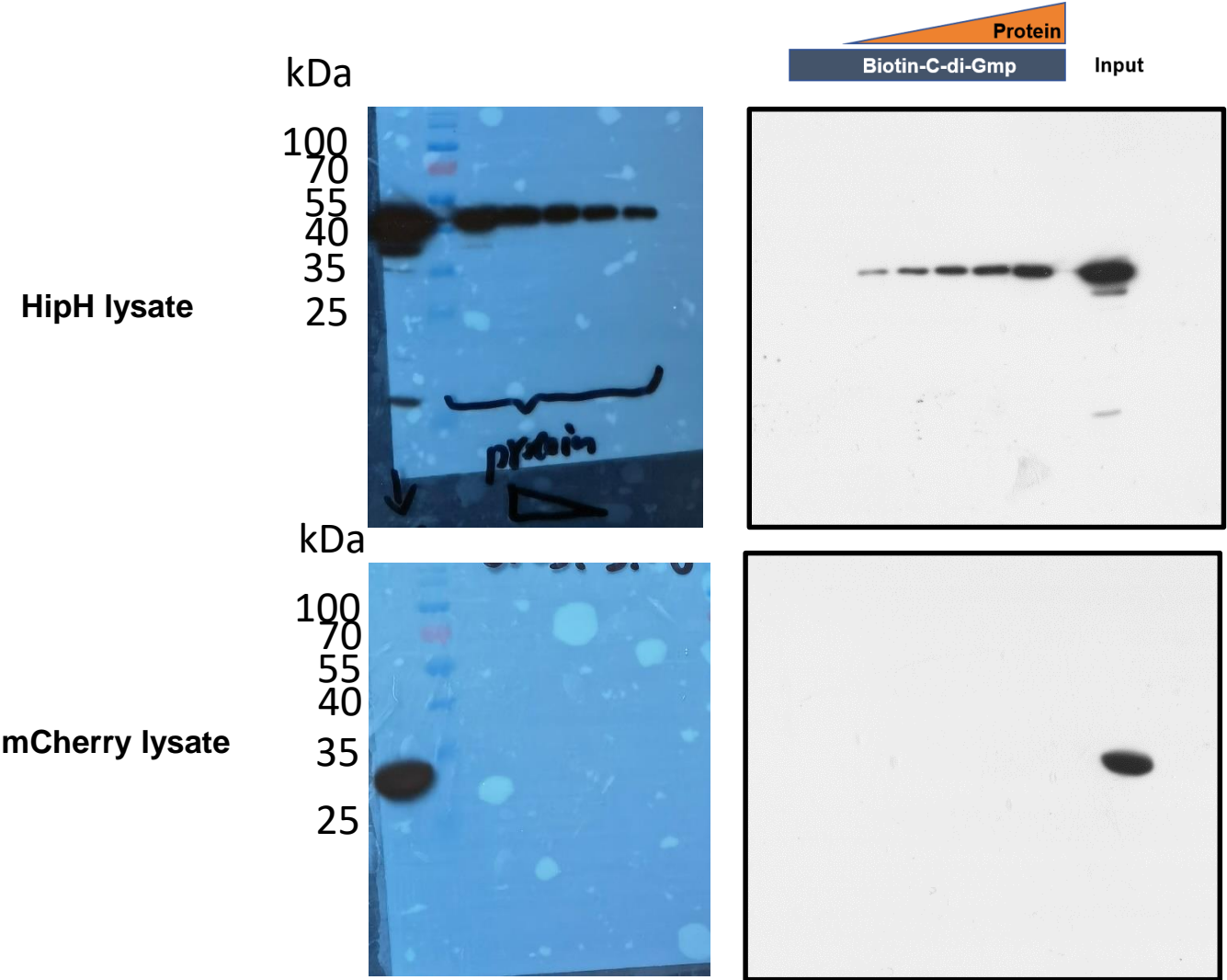

Figure 5—figure supplement 1B

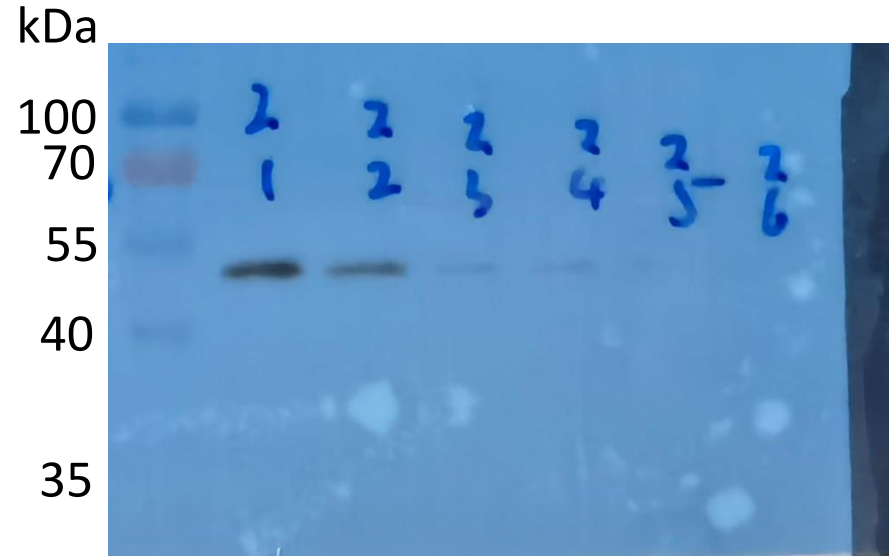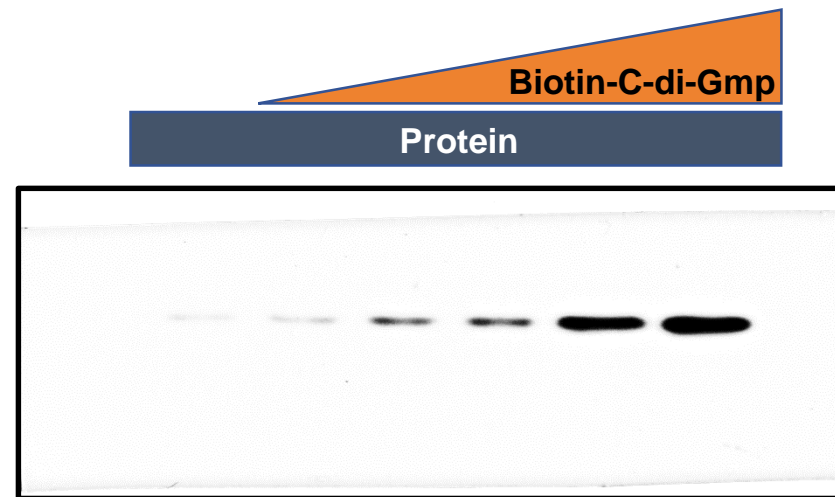

Supplement: Figure 5—figure supplement 1—source data 1. [file elife-99194-fig5-figsupp1-data1.pdf]
